# 

**Published:** 1891-06-27

**Authors:** 


### NOTICE TO CORRESPONDENTS.

All MS., Letters, Books for Review, and other matters intended for the Editor should be addressed The Editor, The Lodge, Porchester Square, London, W.

The Editor cannot undertake to return rejected M.S., even when accompanied by stamped directed envelope.

### Notice to Advertisers and Subscribers.

All Advertisements, Orders for Copies of The Hospital, and Business Communications should be addressed to The Publisher, *not to the Editor*, The Hospital, 140, Strand, W.C.

The Cost of Subscription, post free, is as follows:—Per week, 1½d.; per quarter, 1s. 8d.; per half-year, 3s. 3d.; per annum, 6s. 6d. Foreign:—Per annum, 8s. 8d.; payable, in all cases, in advance.

**NOTICE.—The Index for Vol. IX., (ending March, 1891) is on sale at the Office of this Journal, price 2½d., post free.**

### General Charities.

[This Column is devoted to an account of work of charitable institutions other than Medical Charities. The Editor will be glad to receive reports or news of work at 140, Strand. *Philanthropy* should be plainly written on the envelope.]

The Duke of Edinburgh, presiding at the annual meeting of **THE ROYAL BRITISH FEMALE ORPHAN ASYLUM** in Devonport last week, drew attention to the amount of good which the institution is doing for the orphans of those who have lost their lives in the service of the country. The society is mainly supported by annual subscriptions, which, we regret to learn, are not as numerous as the well-wishers of the institution desire, and which need to be largely increased in order to enable the Committee of Management to carry on the work in an efficient manner.

We are very glad to see an increase in the subscription list of the **FACTORY GIRLS' COUNTRY HOLIDAY FUND.** One hundred and twenty women and girls were sent away last year, and it is to be hoped that more will be sent away this summer. The fund is a branch of the "Women's Help Society," and any information will be gladly given by Miss Canney, St. Peter's Rectory, Saffron Hill. The women and girls contribute to the expense of their trip into the country as much as lies in their power, but there is often a little difficulty about procuring decent clothes, particularly underclothes for the use of the girls, and any help in this way will be valued as much as gifts of money.

One of the most interesting exhibits in the Royal Naval Exhibition is the North Sea trawler, "Heroine," purchased by the **MISSION TO DEEP SEA FISHERMEN** at the close of its final voyage in January last. The brave old vessel has been engaged in the deep sea fishing for over thirty years. When she started on her career in 1858 the lot of the men was rough indeed. Accidents, &c., of various kinds, often serious, were frequent, and the men often suffered untold agony, and sometimes permanent disablement, from want of prompt and skilled assistance; but the society has vastly improved the state of things. They have ten vessels engaged in the service of the mission, and another building. Three of these are properly fitted with hospital accommodation, and carry a qualified medical man, and all the vessels have skippers trained to render first aid to the injured. The Mission to Deep Sea Fishermen hopes to hold a sale of work at Yarmouth in August, when one of the principal features will be a stall furnished by the wives of the fishermen, who, anxious to show their appreciation of the value of the mission to their husbands at sea, are making various articles that could be sold for the benefit of the funds. Articles of every kind suitable for a sale will be gratefully received by A. Gordon, Esq., 181, Queen Victoria Street, London.

The **ASSOCIATION FOR PROMOTING THE GENERAL WELFARE OF THE BLIND**, Berners Street, issues a special appeal for funds to enable the committee to enlarge their premises. The Association, which was started for teaching trades to the blind, employs sixty-seven men and women in their workshops and at their own homes, and twenty-seven others are receiving small pensions; but the objects of the Institution being primarily industrial, the Committee consider that the employés of the Association have a prior claim for pensions over others. The amount paid in wages to the blind is upwards of £2,000 a-year. The inmates are taught basket and brush making, chair caning, and wood chopping; and the prices for household brushes, wooden pails, and mats, &c., are really moderate, and it is by buying these things from the blind that kind-hearted people can help the work forward. The premises are wanted in order to enable more workers to come in, and to bring the articles made by the blind before the notice of the public. We appeal for help to this charity on the ground that there is no greater affliction than blindness, and that this Institution (founded by a blind lady), by enabling blind people to support themselves by useful trades, wages indirectly a war against that odious system which we see carried into effect all over London of gaining a subsistence by the parade of affliction to the ever-sympathetic public.

The **PRINCESS MARY VILLAGE HOMES** at Addlestone, which were founded by Mrs. Meredith in 1871 for the reception of the female children of persons convicted of crime, like most other charities would be very glad of some more money. The Homes are really "homes," and deserve notice from the fact that they are on a small scale, and like cottages in every respect. At the head of each cottage is a mother; the baby and the grown-up daughter who helps to nurse and clean up are the family elements next in importance to the mother, and the remaining eight children all fill their respective places. But this is the point which we draw attention to—the cottages are without the usual facilities and accommodation afforded by large apartments, expensive fittings, and materials of a kind that would be unlikely to fall to the lot of the children in after life so the children are encouraged to contrive and do their best with the simplest household goods. This is far better than bringing up the children with the knowledge that everything is provided for their use in every way, as it makes them use their ingenuity and conduces to their interest in helping to keep up the comfort of the home. This is a lesson that future promoters of the "group" system might reflect on with advantage; it would considerably reduce the bill for "fittings" in any new establishment. The girls trained at Addlestone all go out into domestic service.

### Scraps and Gleanings.

The French Chamber has voted a credit of £60,000 for the destruction of locusts in Algeria.

A concert was given at St. James's Hall on the 12th in aid of the funds of the Italian Hospital in London.

Some handsome subscriptions have been promised towards a cottage hospital for the Rhondda Valley.

Sir Saville Crossley, M.P., has sent the Lord Mayor a donation of £1,000 towards the Hospital Sunday Fund.

A general meeting of the Southern Counties branch of the British Dental Association was held in Hastings on Saturday.

A public meeting was held in Ripley, on the 16th inst., in aid of the fund for the erection of the new Derbyshire Infirmary.

Mr. Applegate, M.R.C.S. Eng. and L.R.C.P. Lond., has been appointed house surgeon at Dewsbury and District Infirmary.

The Marchioness of Salisbury will open the Victoria Hospital at Hull on July 22nd, and she will also launch Her Majesty's ship *Endymion.*

A grand morning concert was given in St. James's Hall on the 17th inst., in aid of the Ladies' Building Fund of the Great Northern Central Hospital.

The foundation stone of the Sick Poor Hospital at Dundee, will be laid early in September. It is proposed to make the ceremony the occasion of a grand demonstration.

Mrs. French Sheldon, who last March left Zanzibar with the intention of visiting Masailand and Mount Kilima Njaro, is now on her way home. She is said to be very weak and ill.

The friendly societies of the districts of Charmouth Rousdon, Uplyme, and Axmouth held a grand parade in aid of the Cottage Hospital. They collected the sum of £7 5s. 10d.

A whole family have been poisoned in Decize, through a servant girl having accidentally gathered cowbane, a species of hemlock, instead of parsley, and mixed it in a dish of haricot beans.

A dramatic performance will be given on the evenings of June 27th, 29th, and 30th, at 25, Pont Street, by permission of Lady Alexandra Gordon-Lennox, in aid of the St. Elizabeth's Hospital.

The Committee of the Zenana Medical College have resolved after the recent census of India, to extend the work in a manner, not hitherto tried, by appointing an organising, or travelling, secretary.

The report for 1890 of the Crewkerne Hospital states that the number of in-patients during the year was 82. The receipts amounted to £696 12s. 7d., and the expenditure to £533 19s. 1½d., leaving a balance in hand of £162 13s. 5½d.

The report for 1890 of the Abingdon Cottage Hospital states that 125 patients were admitted during the year, each patient remaining in the hospital on an average 23 days. The receipts amounted to £633 17s., and the expenditure to £421 16s. 4d.

A Hospital Sunday has been held at Even Swindon, by the joint efforts of the Loyal Great Western Lodge of Odd Fellows and Court "Perseverance," A.O.F. This is the first affair of the kind that has ever been held in Even Swindon.

The collections on Hospital Sunday at St. Mary Abbots, Kensington, amounted to £518 19s. 2d.; at St. Paul's, Knightsbridge, to £422 3s. 8d.; and at Holy Trinity, Paddington, to £228 5s. 2d.

The cross, which has been prepared as a memorial to Father Damien, at the expense of the National Leprosy Fund, is now completed. Mr. H. R. Armstrong, of Messrs. Skinner and Co., has undertaken to ship the memorial free of cost to Molokai, where the Hawaiian Government have promised to have it erected over the grave of Father Damien.

A scholarship, affording free education at the Edinburgh School of Medicine for Women and at Leith Hospital, is offered by a lady to a suitable candidate wishing to devote herself to work as a medical missionary. Candidates are to send in their names to the Secretary of the school, and the selection will be made by a committee of ladies.

The Earl of Aberdeen, accompanied by Lady Aberdeen, presided at the annual meeting of the Invalid Children's Aid Association, which was held in Kensington Town Hall on the 18th inst. During the year 327 new cases have been dealt with, and up to December last 1,077 children had been treated. The Chairman announced that Princess Victoria of Teck had promised to become patron of the Association.

Under the auspices of the London Vegetarian Society, a public debate took place on the 18th inst. at the Memorial Hall, Farringdon Street, on the question, "Is the Temperance Movement a Failure?" The members of the Society maintained vegetarianism was the final solution of the drink question. The speakers were pretty evenly divided, with a small majority opposed to the views of the Society.

The owners of butchers' slaughter-houses in the metropolis, of which there are over 700, are taking action with a view to the insertion of a clause in the Public Health (London) Bill, giving an appeal to the Local Government Board, in the event of the abolition of a slaughter-house licence by the County Council. Lord Carmarthen, Mr. Blundell Maple, and other metropolitan members are interesting themselves in the matter.

At a meeting of the Dunfermline District Committee of the Fife County Council, Dr. Nasmyth suggested that, as a temporary arrangement, a patent portable wooden hospital should be provided, the cost of which was £400, exclusive of furnishings. In moving the adoption of this recommendation, the Earl of Elgin said this hospital would be an adjunct of a permanent hospital, and could be erected in a day or two. The motion was unanimously passed.

At a meeting of those interested in the foundation of a memorial to the late Dr. Matthews Duncan, which was held at St. Bartholomew's Hospital on the 18th inst., it was resolved that a gold medal or other prize for proficiency in obstetrics and gynæcology be founded at St. Bartholomew's Hospital to perpetuate the memory of Dr. Matthews Duncan, to be named the "Matthews Duncan Medal," or Prize. About £1,200 or £1,400 would be required to carry out the memorial.

### BOOKS, &C., RECEIVED.

LEDGER, SMITH, AND CO.

"The Medical Digest, or Busy Practitioners' Vade-Mecum." (Third edition.) R. Neale, M.D. Lond.

H. K. LEWIS.

"Diphtheria: Its Nature and Treatment." (Third Edition.) R. W. Parker.

"Lewis's Pocket Medical Vocabulary." (Second Edition.)

"Intubation of the Larynx." James B. Ball, M.D.

E. AND S. LIVINGSTONE.

Catechism Series: "Anatomy: Abdomen;" and "Medico-Legal Experience in Calcutta." (Price 1s. each).

"Zoology Notes for Students of Medicine and Science." Alexander Johnstone, F G.S. (Price 3s. 6d.)

"Botany Notes for Students of Medicine and Science." Alexander Johnstone, F.G.S. (Price 2s.)

"THE NIGHTINGALE" PUBLISHING COMPANY (New York).

"Massage: A Primer for Nurses." (Second Edition.) Sarah E. Post, M.D. (Price one dollar.)

YOUNG J. PENTLAND.

"Milk Mischief." R. Taylor Manson, L.R.C.P. Edin., M.R.C.S. Eng.

THE VEGETARIAN SOCIETY (Manchester).

"Foods for Man: Animal and Vegetable. A Comparison." Benjamin Ward Richardson, M.D., F.R.S. (Price 3d

J. WRIGHT AND CO. (Bristol).

"Our Unseen Foes, and How to Meet Them. Plain Word son Germ in Relation to Disease." A. Wheeler.

### The Student of Medicine.

[All communications for this Supplement should be addressed to The Editor of The Hospital, at 140, Strand, with the words, "Students' Column," written in the left hand top corner of the envelope.]

### EDITORIAL NOTES.

Since our last issue we have received several communications concerning the fellowship examinations, only endorsing our remarks about the method of conduction of that examination. Examples of some very curious questions that have been asked candidates have also come to hand, in many cases showing the examiners' extraordinary ideas of the subjects they were examining in. We remember very well being somewhat perplexed in the vivâ not many years ago, by being shown a plate containing a piece of beef steak on it and a glass of milk by the side, and were asked what it was? What answer the learned physiologist required we have never to this day determined. But we have heard of one candidate who, on being asked the same problem, suggested, "Your lunch, sir." So also we were shown an egg, and asked what that was? The examiner, not being satisfied with our answer to these vital points, next requested us to look down a microscope at a section, under about a sixth of an inch power, and on attempting to bring it into focus with fine adjustment, we were requested in rather sharp tones not to touch that, as we did not understand it. Having been for some time demonstrator in physiology in one of the leading Universities, and done a considerable amount of microscopic work, needless to say, we were rather taken aback, and not a little disgusted. Among the specimens shown was that of a liver, and the examiner seemed very annoyed because we did not recognise it as that from a polar bear.

A point we have failed to understand is why the candidates for the fellowship examination are not examined in Latin. Why should the education of the hospital surgeons be so unlike that of a gentleman as compared with the curriculum of the hospital physician? A man to be permitted to present himself as a candidate for the appointment of physician to most of the hospitals has had to pass a severe examination at the Royal College of Physicians, where, as has been said, "his knowledge of medicine is judged by whether he can translate easy passages from Celsius." Why then should the surgeons' standard of education be so very different from the physicians'? The two Royal Colleges have now become amalgamated, and it is most painful to think that the surgeons have not adhered to the ancient so-called Latin superstition of Huxley, when the physicians have. Or, if the physicians' easy passages of Latin are too difficult, why not examine in still easier passages of Greek? This would really be an opening for them to show how smart they are. And we await the introduction of Xenophon with time.

To show the extreme value of Latin to the profession at large, and especially to teachers, the following experience may be instructive. We were going round the wards of one of the larger hospitals the other day, in the vain hope of learning something or of picking up some crumbs from the physician in charge of the patients. Our mind was beginning to wander, as we had ceased to expect to hear a diagnosis suggested, when after for some moments contemplating a serious case, the following prescription was dictated by the teacher: Haustus Sennæ Compositæ, unciam unam, pro re natâ. A student standing behind us eager for knowledge and having grasped the dose of Haust Sennae Co., asked his neighbour, what was the dose of pro re natâ.

### APPOINTMENTS.

At the Blackburn and East Lancashire Infirmary, W. A. Bryant, M.B., C.M.Edin., has been appointed junior house surgeon, and W. Harting Bunting senior house surgeon. At the West Norfolk and Lynn Hospital, G. R. Chadwick, M.R.C.S., L.S.A., has been appointed honorary surgeon. At the Farringdon General Dispensary, Charles William Chapman, M.D.Durh., M.R.C.P.Lond., has been appointed honorary physician. At the Royal South

### THE STUDENT OF MEDICINE—(*continued*).

London Dispensary, J. V. C. Denning, L.K.Q.C.P., L.R.C.S. Irel., has been appointed honorary surgeon. At the South Western Fever Hospital, T. A. M. Ford, L.R.C.P., M.R.C.S., has been appointed resident clinical assistant. At the Sheffield School of Medicine, A. J. Hall, B.A., M.B.Cantab., L.R.C.P. Lond., has been appointed joint lecturer on physiology, and demonstrator of practical physiology. At the Hull Royal Infirmary, E. Harrison, M.A., M.B.Cantab., F.R.C.S.Eng., has been appointed honorary assistant surgeon, E. H. Howlett, F.R.C.S. Eng., L.R.C.P.Lond., honorary assistant surgeon, also D. Lowson, M.D.Aberd., M.R.C.S.Eng., honorary assistant surgeon; H. W. Pigeon, M.A.M.B., F.R.C.S.Eng., honorary assistant surgeon; and W. C. Rockliffe, M.A., M.D., M.R.C.S.Eng., re-appointed ophthalmic surgeon. At the Royal Hospital for Diseases of the Chest, City Road, E.C., M. A. Khan, M.R.C.S., L.R.C.P., has been appointed house physician. R. Herron, L.R.C.S.Irel., L.R.C.P.Edin., has been appointed medical officer and consulting sanitary officer to the Workhouse Armagh Union. At the Halifax Infirmary End Dispensary, Lucius MacDonnell, B.A., M.B., B.Ch., B.A.O., has been appointed assistant house surgeon. At the Royal Berks Hospital, Reading, Charles W. Marriott, M.D., M.R.C.P.Lond., has been appointed physician. At the Aberdeen Royal Infirmary, J. Scott Riddell, M.B., C.M., M.A.Aberd., has been appointed assistant pathologist. At the Sussex County Hospital, Brighton, F. Mortimer Rowland, M.A., M.R.C.S., L.R.C.P.Lond., has been appointed assistant house surgeon. At the General Hospital, Birmingham, Thomas S. Short, M.B.Lond., M.R.C.S., has been appointed honorary assistant physician. At the Rotherham Hospital and Dispensary, Robert Arthur Smith has been appointed house surgeon. At the Cheltenham Children's Hospital, Herbert Ward-Humphreys, L.R.C.P., M.R.C.S., has been appointed surgeon. At the Dewsbury and District Infirmary, J. W. Applegate. L.R.C.P., M.R.C.S., has been appointed house surgeon. At the Plymouth Borough Lunatic Asylum, A. N. Davis, L.R.C.P., L.R.C.S.Edin., has been appointed medical superintendent. At the Royal Lunatic Asylum, Montrose, James C. Howden, M.D.Edin., has been appointed medical superintendent. At the Royal Infirmary, Aberdeen, Alex. Macgreggor, M.B., C.M., has been appointed electrician. At the Mater Misericordiæ Hospital, Dublin, M. C. Staunton, M.B., C.M.Irel., has been appointed house surgeon.

### VACANCIES.

The following vacancies are announced this week, the amount of salary in each case being given after the name of the institution:

**Resident Medical Officers.**

Baltinglass Union, Medical Officer for the Workhouse and Fever Hospital—Salary £120.
Bedford General Infirmary and Fever Hospital, Resident Surgeon—Salary £100, with board, &c.
County Donegal Infirmary, House Surgeon and Registrar—Salary, £60.
Derbyshire Royal Infirmary, Resident Assistant House Surgeon—Salary £10, for six months.
General Hospital, Birmingham. Assistant House Surgeon—Board, &c.
General Infirmary at Gloucester and Gloucestershire Eye Infirmary, House Surgeon—Salary £100, with board, &c.
General Infirmary, Leeds, Resident Medical Officer—Salary £100, with board, &c.; also Resident Casualty Officer — Salary £100, with board, &c.
Hospital for Consumption and Diseases of the Chest, Brompton, House Physicians—Board, &c.
Lunatic Asylum, The Coppice, Nottingham, Assistant Medical Officer, Salary £100, with board, &c.
North-West London Hospital, Assistant Resident Medical Officer—Board, &c.
Wandsworth and Clapham Infirmary, St. John's Hill, S.W., Junior Assistant Resident Medical Officer—Salary £25 for six months.

**Non-Resident Medical Officers.**

Chelsea Hospital for Women, Fulham Road, Anæsthetist.
General Hospital, Birmingham, Assistant Surgeon.
Guy's Hospital, five Assistant Dental Surgeons.
Queen's College, Birmingham, Medical Tutor and Demonstrator of Anatomy, also a Demonstrator of Anatomy.
Royal Westminster Ophthalmic Hospital, Clinical Assistants.
University of Glasgow, Examiner in Physiology—Annual fee 30 guineas.
Victoria Hospital for Sick Children, Physician to the in-patients; also Physician to the out-patients.
Victoria University, Holt Professorship of Physiology.

### NOTES FROM THE SCHOOLS.

**Cambridge University.**

The following degrees have been conferred:—

Doctors of Medicine. — Lawrence Humphry, Trinity; William Arthur Foxwell, St. John's; Charles Eardley Dumbleton, Peterhouse;

### THE STUDENT OF MEDICINE—(*continued*).

Walter Knowsley Sibley, Pembroke; George Fuller Ashbridge England, Walter Horace Goddard, Gonville and Caius; George Hutchinson Milnes, Downing.

DOCTORS OF SCIENCE.—William Mitchinson Hicks, St. John's; John Neville Keynes, Pembroke.

BACHELOR OF MEDICINE.—Ernest Henry Richmond Watts, St. John's.

BACHELORS OF MEDICINE AND BACHELORS OF SURGERY.—Andrew Hallidie Smith Hallidie, King's; Stanley Lewis, St. John's; William Robson Carter, Robers Hardwick Hall, Cecil Latter, Pembroke; Hugh Kerr Anderson, Charles Simmons Bennetts, Gordon Calthrop, Herbert Thomas Crosby, James Lea, Herbert William Nix, John Charles Ryder Richardson, Edward Ernest North Surridge, Joseph Henry Wilks, Gonville and Caius; Alexander Miller, Queen's; Stamford Grant Felce, Jesus; George Wilkinson, Emmanuel; William Lawrence Woodward Buss, Sidney Sussex; William Henry Beaumont, Charles Stuart Pethick, Albert Francis Richards, Downing; Robert Stephen Earl, John Fisher, Karl Carwardine Gimson, Charles Gordon Roberts, Horace Savory, Cavendish Hostel.

### SCHOLARSHIPS AND PRIZES.

### University of Cambridge.

At a congregation held on June 4th, Professor Foster was appointed the representative of the University on the Council of the Marine Biological Association. Also the following degrees were conferred: Doctor of Medicine, Francis Frederick Schacht, Trinity; Bachelor of Medicine and Bachelor of Surgery, Cyril Henry Cayley, Pembroke.

### PASS LISTS.

### Cambridge University.

SECOND EXAMINATION FOR MEDICAL AND SURGICAL DEGREES. EASTER TERM, 1891.

PART I.—PHARMACEUTICAL CHEMISTRY.

EXAMINED AND APPROVED: Bond, B.A., non-collegiate; Bowring, Cai.; W. L. Brown, Joh.; Bumsted, B.A., Joh.; Burnett, Joh.; Cundy, M.A., Trin.; Dashwood, Magd.; C. D. Edwards, Joh.; Evans, Trin.; F. A. Godson, Joh.; R. J. E. Hanson, Trin.; Hey, Trin.; Hutchinson, B.A., Trin. H.; Hyde, B.A., Clare; T. L. Jackson, Joh.; Lance, King's; Leach, non-collegiate; Lowe, Jesus; Maturin, Cai.; McCardie, Cai.; Moore, B.A., Joh.; Muriel, Down, Myers, Cai.; Pearce, Trin.; J. A. K. Renshaw, Trin.; Robinson, Emman.; Russell, B.A., Emman.; Salt, Cai.; Sedgwick, Clare; Seligman, B.A., Cai.; Sheppard, Christ's; J. Smith, Jesus; Stead, Cai.; C. B. Stewart, M.A., Christ's; K. S. Storrs, Emman.; Thomson, Christ's; Thorley, B.A., Clare; Tyrrell, Clare; Vernon, Jesus; F. J. Watson, Trin.; H. F. B. Williams, B.A., Cai.; Wills, Cai.

NATURAL SCIENCES TRIPOS, PART I., 1891.

CLASS I.—Brownsword, Down; Burkill, Cai.; Colby, King's; A. R. Cook, Trin.; Eichholz, Emman.; Keeble, Cai.; D. H. Moore, Trin.; Murphy, Cai.; Reed, Trin.; Taylor, Emman.; Todd, Clare; Villy, Joh.; B. W. Watson, Clare; Widdicombe, Down.

CLASS II.—Bennett, B.A., Queens'; Bennett, Joh.; Blumfeld, Cai.; Bowes, Cai.; Burton, King's; J. A. Cameron, Joh.; Cheadle, Trin.; R. Crosthwaite, B.A., Pemb.; Denmead, B.A., Emm.; Gardiner, B.A., Chr.; Goodman, Joh.; Guinness, Cai.; W. J. Harris, Cai.; Harris, Cla.; Hayward, Cla.; Henderson, Joh.; Hunter, H. Cav.; Hutchings, B.A., H. Selw.; Killick, Trin.; Laurie, King's; M'Carthy, Cai.; G. J. K. Martyn, Cai.; Mathew, Trin. H.; Olliver, Trin.; Ormerod, H. Selw.; Purvis, Joh.; Richards, Sid.; Sandall, Joh.; Sell, Cai.; Swift, Down; Tripp, B.A., Cai.; Trotman, Joh.; E. F. C. Ward, Sid.; Weller, Chr.; R. G. Wrightson, Trin.

CLASS III.—Allen, King's; Atkinson, B.A., Trin.; Barclay, Trin. H.; Bartlett, non-collegiate; Bates, Queens'; Bellhouse, Trin.; Berry, King's; Blaydes, King's; Boake, Cai.; Borcherds, Cai.; Candler, H. Selw.; Cornwall, Trin.; Cowan, King's; Daniel, Emm.; L. G. Davies, non-collegiate; Fayrer, Trin.; Garner, Emm.; Hayne, Cai.; Hofmeyr, Trin. H.; Hood, H. Cav.; C. L. Hopkins, Cai.; F. A. S. Hutchinson, Trin.; Jesse, H. Selw.; Key, Emm.; T. P. King, Joh.; F. H. Lewis, Joh.; Longfield, B.A., Corp.; Mackenzie, Cai.; C. E. Marriott, Cla.; Marshall, Cai.; Milward, Cla.; Munts, Trin.; Parkinson, Chr.; Ll. Powell, Trin.; M. C. Powell, Trin.; Read, Emm.; Reece, Down; H. N. Sheppard, B.A., Trin.; Sparks, Cai.; Trethewy, Cai.

ALLOWED THE ORDINARY B.A. DEGREE.—J. U. N. Bardsley, Cai.; J. W. Crewdson, Trin.; Hadow, Cai.; Haigh, Joh.; G. C. Jackson, Joh.; H. G. T. Jones, Joh.; Kirk, Chr.; E. B. Marriott, Cla.; Mills, Cla.; Oldfield, Cla.; Pasteur, Trin.; Rotherham, Trin.; Twigg, Chr.

EXCUSED THE GENERAL EXAMINATION FOR B.A. DEGREE.—Hutchinson, Joh.

ÆGROTANT.—J. E. G. Gardiner, Cai.; Locker, Queens'; May, Cla.; Stanton, Magd.

WOMEN.

CLASS I.—E. C. J. Abbot, Newnham.

CLASS II.—S. Annesley, Girton; F. M. Durham, Girton; M. A. Luke, Girton; E. Macdonald, Girton; E. A. Mead, Newnham; E. Wright, Girton.

CLASS III.—B. Clarke, Newnham; A. M. Fletcher, Girton; O. M. S. Lewin, Girton; S. A. Storrar, Newnham; M. Walmsley, Newnham.

### THE STUDENT OF MEDICINE—(*continued*).

Reached the Standard of an Ordinary B.A. Degree.—U. Chaplin, Girton.

Natural Sciences Tripos, Part II., 1891.

Class I.—Blackman, Joh. (botany); Bottomley, (Cai. (human anatomy with physiology); †Capstick, Trin. (chemistry, physics); Krishnan, B.A., Chr. (chemistry, botany); H. Kynastion, King's (geology); Macbride Joh. (zoology, botany); Spivey, Trin. (chemistry); Whichello, Sid. (botany); T. B. Wood, Cai. (chemistry).

Class II.—Collcutt, B.A., Cai.; Cuff, Joh.; Peters, Cai.; Schmitz., B.A., Joh.; T. Manners Smith, B.A.. Down.

Class III.—Clark, B.A., Sid.; Grünbaum, Cai.; Theobald, B.A., Joh.; Troup, B.A., Pemb.; Whiteley, B A.

Ægrotant.- Bottomley, King's; Herbert, King's.

† Distinguished in chemistry and in physics.

Women.

Class I.—A. I. M. Elliot, Newnham (zoology); M. C. Tebb, Girton (physiology).

Class II.—E. Dale, Girton; H. M. Martyn, Newnham.

Class III.—S. A. Gardiner, Newnham; F. G. Whitting, Newnham.

### Royal College of Surgeons of England.

The following gentlemen having passed the necessary examinations, and having conformed to the bye-laws and regulations, were, at an ordinary meeting of the Council on Thursday, June 11th, admitted Fellows of the College: Pronger, Charles Ernest, L.R.C.P Lond., South Kensington; Diploma of Member dated Jan. 27th, 1876. Morley, Edward John, L.R.C.P.Lond., Chatham; January 21st, 1879. Nance, Henry Chester, L.R.C.P.Lond., Norwich; May 18th, 1880. Turton, James, L.S.A., Brighton; July 29th, 1880. Wallace, F. C., M.B. Cantab, St. James Street, W.; Jan. 25th, 1883. Hughes, A. W., L.R.C.P.Lond., Musselburgh, N.B.; July 23rd, 1885. Rayner, Herbert Edw., L.R.C.P. Lond., Maida Vale; Oct. 23rd, 1885. Herbert, Herbert, L.R.C.P.Lond., Bombay Army; April 19th, 1886. Stocks, William Percy, L.S.A., Salford; Nov. 10th, 1887. Heaton, George, L.R.C.P.Lond., Birmingham; Feb. 9th, 1888. Lucas, Albert, L.R.C.P.Lond., Bedford; Feb. 9th, 1888. Teichelmann, Ebenezer, L.K.Q.C P.I., Birmingham; Feb. 9th, 1888. Roberts, John Lloyd, L.R.C.P Lond., Colchester; May 10th, 1888. Housman, Basil Williams, L R.C.P Lond., Taunton; Aug. 2nd, 1888. Campbell, John, M.D.R.U.Irel., Belfast; Nov. 8th, 1888. Macgregor, Jos. Johnstone, M.B.Lond., Royal Orthopædic Hospital; May 9th, 1889. Cobbett, Louis, L.R.C.P.Lond., Weybridge; Feb. 13th, 1890; Blacker, Geo. Francis, L.R.C P.Lond., Blackheath; July 28th, 1890. Johnstone, James, M.B.Aberd., Manchester; July 28th. 1890. Kellock, T. H., L.R.C.P.Lond., Lambeth Palace-road; Feb. 12th, 1891. Ross, Daniel McClure, Upper Berkeley-street, W.; not a member. Dyall, Thos. James, M.B.Lond., Hackney-road, N.E.; not a member. Seven other candidates passed the examinations, but not yet having attained the legal age (twenty-five years) will receive their diplomas at future meetings of the Council. Thirteen candidates were referred.

The following gentleman having passed the necessary examinations, and having conformed to the by-laws and regulations, was at the same meeting admitted a member: Bates, James Curling, L.R.C.P.Lond., West Norwood.

The following gentlemen having passed the necessary examinations, and having conformed to the by-laws and regulations, were admitted Licentiates in Dental Surgery: Allin, Charles James, St. Alban's; Badcock, Christopher Frampton, Tottenham Court Road, W.; Birkett, William, Morecambe, Lancs.; Blaaberg, Charles Jens, Argyle Square, King's Cross; Breese, Frederick, Crouch Hill, N.; Coysh, Thomas Arthur, Brixton; Forsyth, Harry Arthur, Claverton Street, S.W.; Gask, Arthur Cecil, West Kensington; Harrison, Richard Malcolm Crowth r, Camden Road, N.; Haynes, Frederick, Southsea: Headridge, David, Manchester; Hopson, Montagu Frank, Newbury; Jones, David Richard, Swansea; Longhurst, Percy Augustus, Barnes Common; Stocken, Leslie Maury, M.R.C.S. Eng., Uxbridge Road, W.; Walker, Frederick Vincent, Doncaster; Welham, George William, Brixton Road, S.W. Seventeen candidates were referred to their professional studies for six months.

---

### ATHLETICS.

### Cricket.—June 13th.

*London Hospital* beat the *Leys School* at Cambridge by 60 runs. For the Hospital Birt made 53. Scores: Hospital. 120; Leys, 60.

*Guy's Hospital* beat *Kensington Park* at St. Quintin's Park, Notting Hill, by 54 runs. Scores: Hospital, 207; Kensington Park, 153.

*University College Hospital* were beaten by *Kensington*, at Eynfields, by an innings and 137 runs. Scores: Kensington, 223; Hospital, 42 and 44.

Inter-Hospital Challenge Cup.—2nd Round.

*St. Mary's v. Charing Cross.*—St. Mary's, 1st innings.—D. D'Esterre, b Wright, 7; B. Pares, c Thomas, b Russell, 5; E. G. Mayo, c and b Russell, 1; W. Overend, b Wright, 1; E. G. Moon, b Wright, 0; E. W. Jocelyne, run out, 4; F. Bond, b Wright, 5; L. Oliver, b Wright, 1; J. Matthews, c Wallace, b Russell, 8; S. G. Fells, c Escombe, b Young, 7; E. J. Poynton, not out, 3; byes, 9; total, 51. Charing Cross, 1st innings.—C. S. Cynen, 1; C. H. Russell, 6; S. N. Wright, 24; W. Escombe, 27; C. W. Lanphier, 4; H. H. Thomas, 5; E. C. Montgomery, 1; W. W. Ashfield, 2; C. J. Young, 0; G. P. Parry, not out, 2; extras, 7; total, 80. St. Mary's, 2nd innings, 72 for five wickets. Innings declared closed. Charing Cross, 42 for one wicket.

